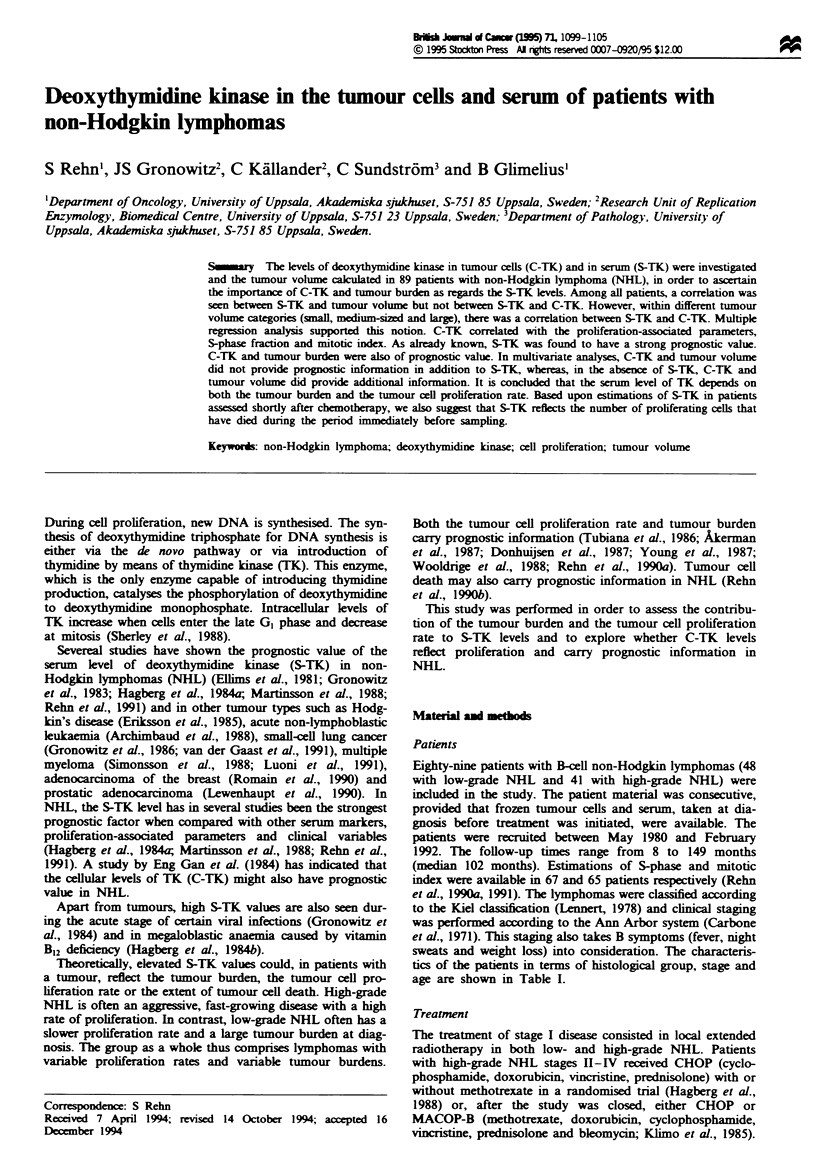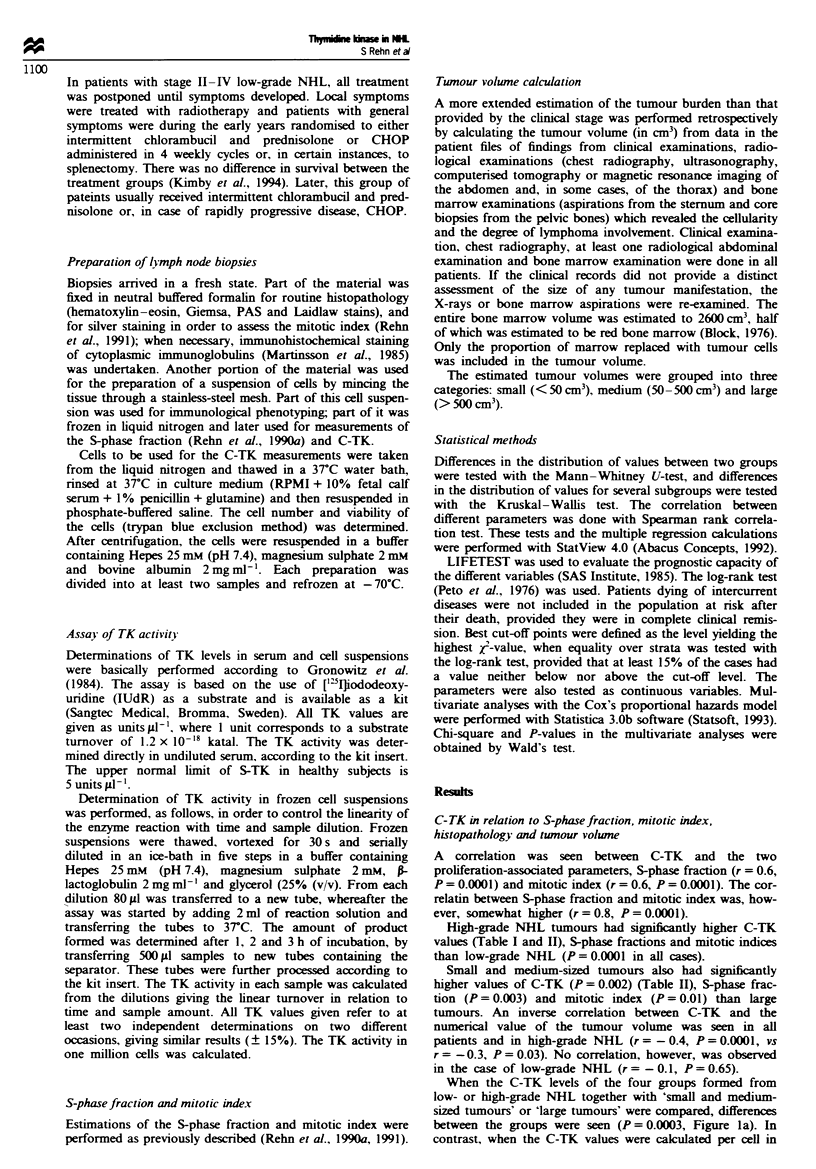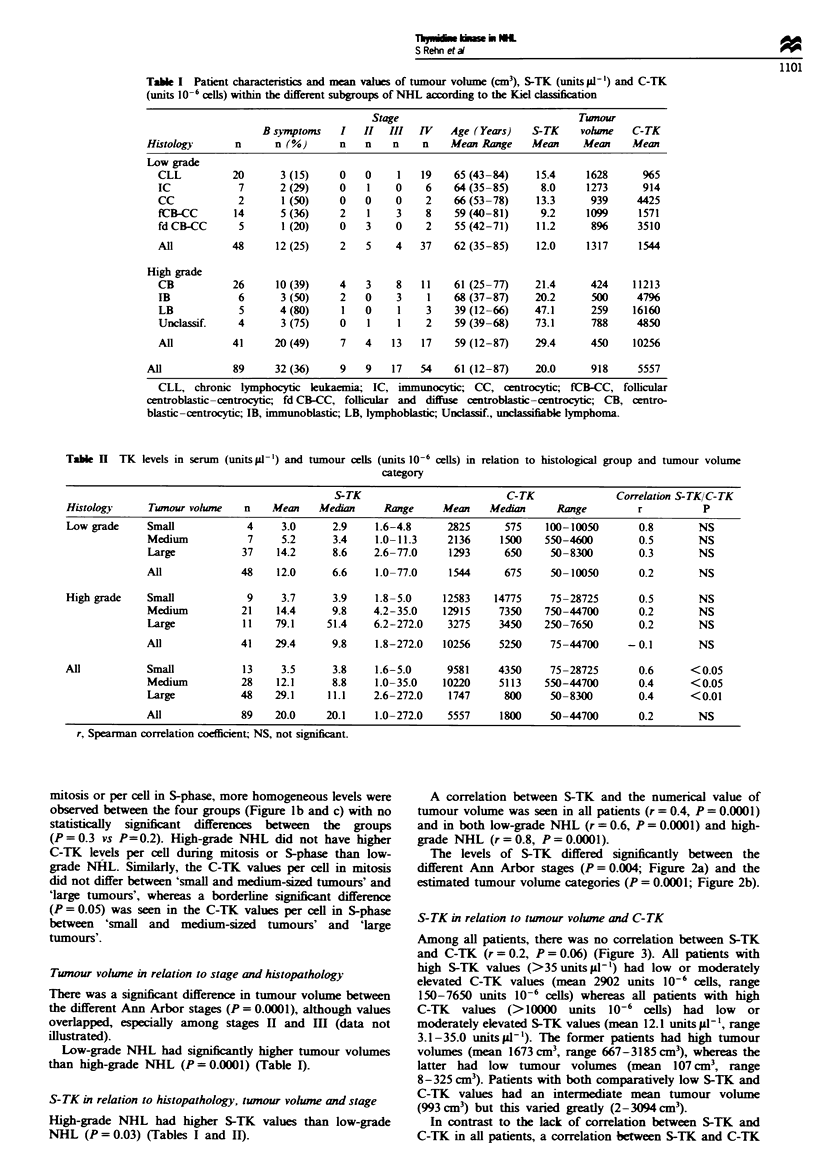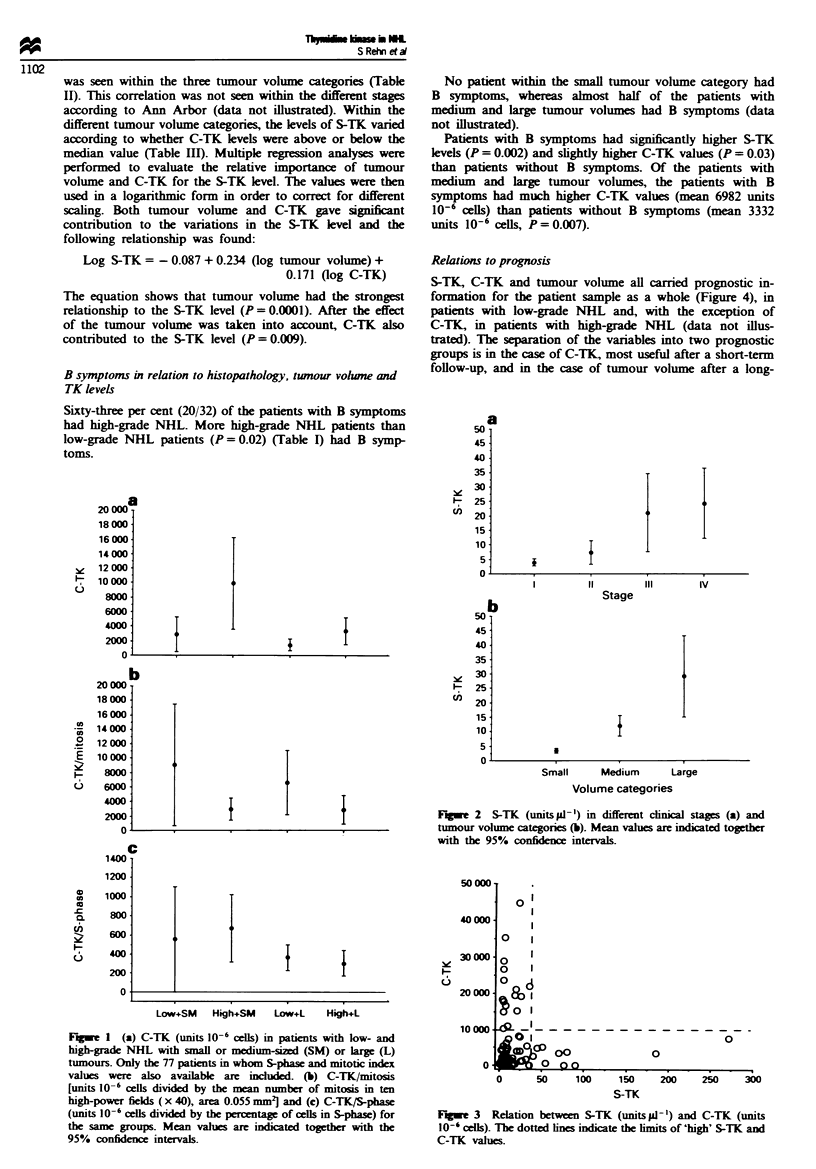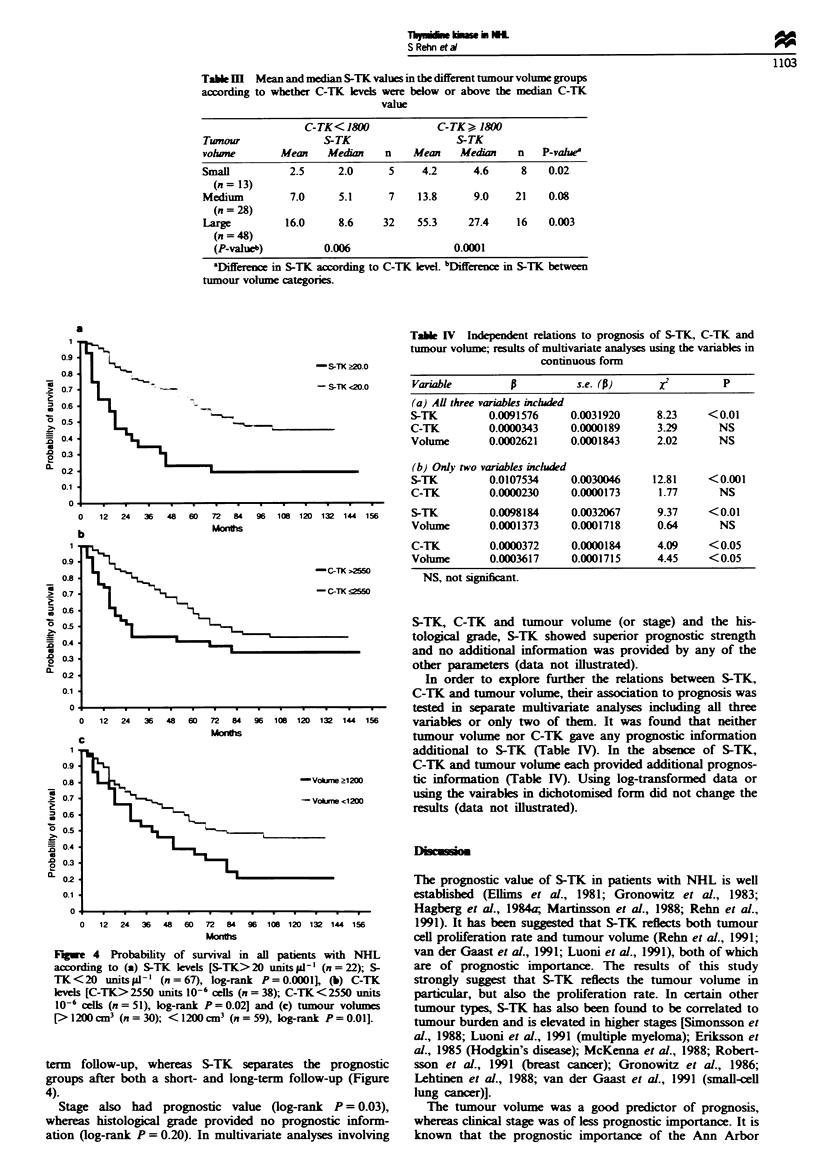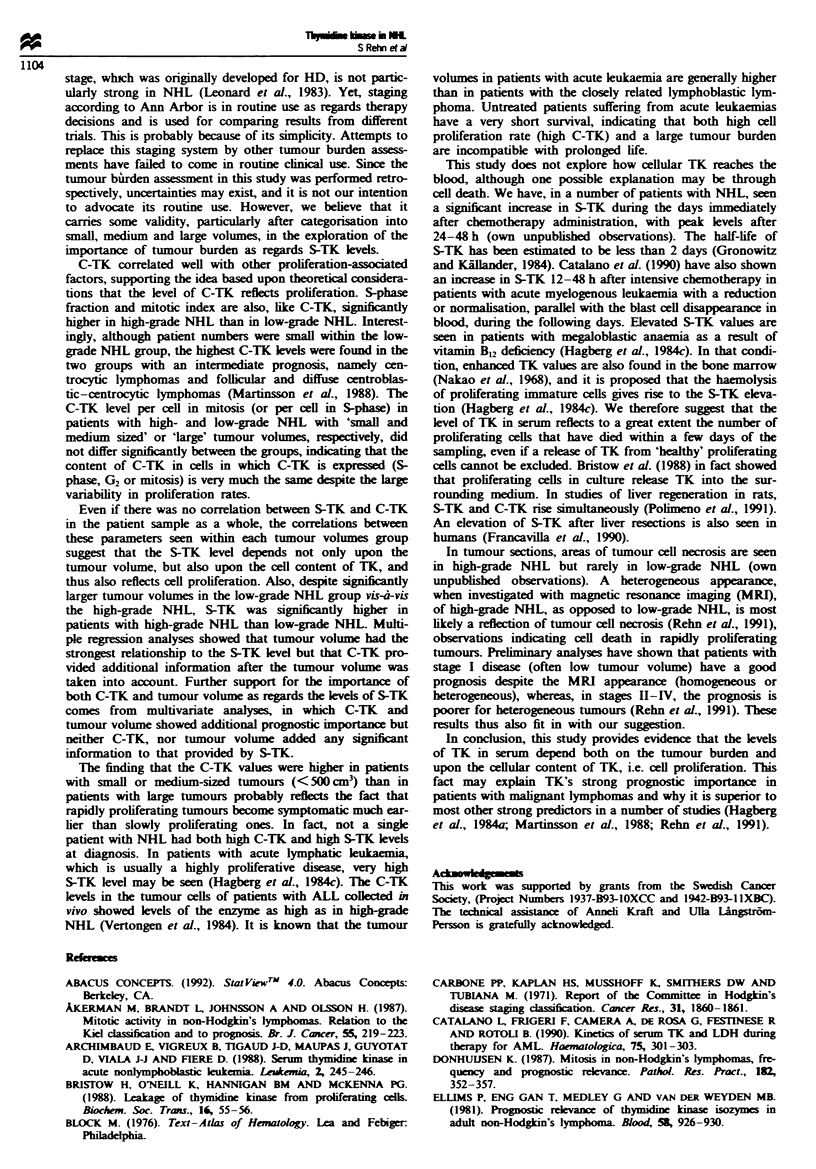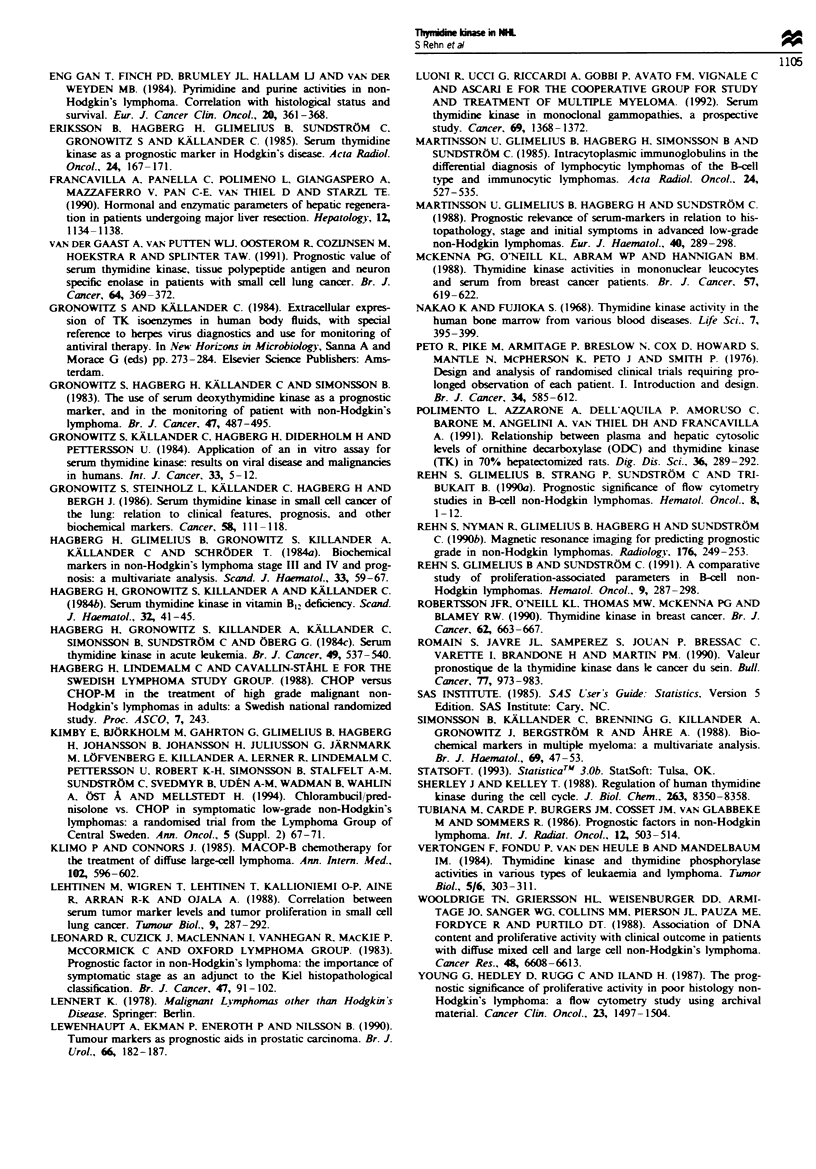# Deoxythymidine kinase in the tumour cells and serum of patients with non-Hodgkin lymphomas.

**DOI:** 10.1038/bjc.1995.213

**Published:** 1995-05

**Authors:** S. Rehn, J. S. Gronowitz, C. Källander, C. Sundström, B. Glimelius

**Affiliations:** Department of Oncology, University of Uppsala, Akademiska sjukhuset, Sweden.

## Abstract

The levels of deoxythymidine kinase in tumour cells (C-TK) and in serum (S-TK) were investigated and the tumour volume calculated in 89 patients with non-Hodgkin lymphoma (NHL), in order to ascertain the importance of C-TK and tumour burden as regards the S-TK levels. Among all patients, a correlation was seen between S-TK and tumour volume but not between S-TK and C-TK. However, within different tumour volume categories (small, medium-sized and large), there was a correlation between S-TK and C-TK. Multiple regression analysis supported this notion. C-TK correlated with the proliferation-associated parameters, S-phase fraction and mitotic index. As already known, S-TK was found to have a strong prognostic value. C-TK and tumour burden were also of prognostic value. In multivariate analyses, C-TK and tumour volume did not provide prognostic information in addition to S-TK, whereas, in the absence of S-TK, C-TK and tumour volume did provide additional information. It is concluded that the serum level of TK depends on both the tumour burden and the tumour cell proliferation rate. Based upon estimations of S-TK in patients assessed shortly after chemotherapy, we also suggest that S-TK reflects the number of proliferating cells that have died during the period immediately before sampling.


© 1995 Stockton Press All rights reserved 0007–0920/95 $12.00

### Deoxythymidine kinase in the tumour cells and serum of patients with non-Hodgkin lymphomas

S Rehn[1], JS Gronowitz[2], C Källander[2], C Sundström[3] and B Glimelius[1]

[1]*Department of Oncology, University of Uppsala, Akademiska sjukhuset, S-751 85 Uppsala, Sweden;* [2]*Research Unit of Replication Enzymology, Biomedical Centre, University of Uppsala, S-751 23 Uppsala, Sweden;* [3]*Department of Pathology, University of Uppsala, Akademiska sjukhuset, S-751 85 Uppsala, Sweden.*

**Summary** The levels of deoxythymidine kinase in tumour cells (C-TK) and in serum (S-TK) were investigated and the tumour volume calculated in 89 patients with non-Hodgkin lymphoma (NHL), in order to ascertain the importance of C-TK and tumour burden as regards the S-TK levels. Among all patients, a correlation was seen between S-TK and tumour volume but not between S-TK and C-TK. However, within different tumour volume categories (small, medium-sized and large), there was a correlation between S-TK and C-TK. Multiple regression analysis supported this notion. C-TK correlated with the proliferation-associated parameters, S-phase fraction and mitotic index. As already known, S-TK was found to have a strong prognostic value. C-TK and tumour burden were also of prognostic value. In multivariate analyses, C-TK and tumour volume did not provide prognostic information in addition to S-TK, whereas, in the absence of S-TK, C-TK and tumour volume did provide additional information. It is concluded that the serum level of TK depends on both the tumour burden and the tumour cell proliferation rate. Based upon estimations of S-TK in patients assessed shortly after chemotherapy, we also suggest that S-TK reflects the number of proliferating cells that have died during the period immediately before sampling.

**Keywords**: non-Hodgkin lymphoma; deoxythymidine kinase; cell proliferation; tumour volume

During cell proliferation, new DNA is synthesised. The synthesis of deoxythymidine triphosphate for DNA synthesis is either via the *de novo* pathway or via introduction of thymidine by means of thymidine kinase (TK). This enzyme, which is the only enzyme capable of introducing thymidine production, catalyses the phosphorylation of deoxythymidine to deoxythymidine monophosphate. Intracellular levels of TK increase when cells enter the late $G_1$ phase and decrease at mitosis (Sherley *et al.*, 1988).

Severeal studies have shown the prognostic value of the serum level of deoxythymidine kinase (S-TK) in non-Hodgkin lymphomas (NHL) (Ellims *et al.*, 1981; Gronowitz *et al.*, 1983; Hagberg *et al.*, 1984*a*; Martinsson *et al.*, 1988; Rehn *et al.*, 1991) and in other tumour types such as Hodgkin's disease (Eriksson *et al.*, 1985), acute non-lymphoblastic leukaemia (Archimbaud *et al.*, 1988), small-cell lung cancer (Gronowitz *et al.*, 1986; van der Gaast *et al.*, 1991), multiple myeloma (Simonsson *et al.*, 1988; Luoni *et al.*, 1991), adenocarcinoma of the breast (Romain *et al.*, 1990) and prostatic adenocarcinoma (Lewenhaupt *et al.*, 1990). In NHL, the S-TK level has in several studies been the strongest prognostic factor when compared with other serum markers, proliferation-associated parameters and clinical variables (Hagberg *et al.*, 1984*a*; Martinsson *et al.*, 1988; Rehn *et al.*, 1991). A study by Eng Gan *et al.* (1984) has indicated that the cellular levels of TK (C-TK) might also have prognostic value in NHL.

Apart from tumours, high S-TK values are also seen during the acute stage of certain viral infections (Gronowitz *et al.*, 1984) and in megaloblastic anaemia caused by vitamin $B_{12}$ deficiency (Hagberg *et al.*, 1984*b*).

Theoretically, elevated S-TK values could, in patients with a tumour, reflect the tumour burden, the tumour cell proliferation rate or the extent of tumour cell death. High-grade NHL is often an aggressive, fast-growing disease with a high rate of proliferation. In contrast, low-grade NHL often has a slower proliferation rate and a large tumour burden at diagnosis. The group as a whole thus comprises lymphomas with variable proliferation rates and variable tumour burdens.

Both the tumour cell proliferation rate and tumour burden carry prognostic information (Tubiana *et al.*, 1986; Åkerman *et al.*, 1987; Donhuijsen *et al.*, 1987; Young *et al.*, 1987; Wooldrige *et al.*, 1988; Rehn *et al.*, 1990*a*). Tumour cell death may also carry prognostic information in NHL (Rehn *et al.*, 1990*b*).

This study was performed in order to assess the contribution of the tumour burden and the tumour cell proliferation rate to S-TK levels and to explore whether C-TK levels reflect proliferation and carry prognostic information in NHL.

### Material and methods

### *Patients*

Eighty-nine patients with B-cell non-Hodgkin lymphomas (48 with low-grade NHL and 41 with high-grade NHL) were included in the study. The patient material was consecutive, provided that frozen tumour cells and serum, taken at diagnosis before treatment was initiated, were available. The patients were recruited between May 1980 and February 1992. The follow-up times range from 8 to 149 months (median 102 months). Estimations of S-phase and mitotic index were available in 67 and 65 patients respectively (Rehn *et al.*, 1990*a*, 1991). The lymphomas were classified according to the Kiel classification (Lennert, 1978) and clinical staging was performed according to the Ann Arbor system (Carbone *et al.*, 1971). This staging also takes B symptoms (fever, night sweats and weight loss) into consideration. The characteristics of the patients in terms of histological group, stage and age are shown in Table I.

### *Treatment*

The treatment of stage I disease consisted in local extended radiotherapy in both low- and high-grade NHL. Patients with high-grade NHL stages II–IV received CHOP (cyclophosphamide, doxorubicin, vincristine, prednisolone) with or without methotrexate in a randomised trial (Hagberg *et al.*, 1988) or, after the study was closed, either CHOP or MACOP-B (methotrexate, doxorubicin, cyclophosphamide, vincristine, prednisolone and bleomycin; Klimo *et al.*, 1985).

Correspondence: S Rehn
Received 7 April 1994; revised 14 October 1994; accepted 16 December 1994

In patients with stage II–IV low-grade NHL, all treatment was postponed until symptoms developed. Local symptoms were treated with radiotherapy and patients with general symptoms were during the early years randomised to either intermittent chlorambucil and prednisolone or CHOP administered in 4 weekly cycles or, in certain instances, to splenectomy. There was no difference in survival between the treatment groups (Kimby *et al.*, 1994). Later, this group of pateints usually received intermittent chlorambucil and prednisolone or, in case of rapidly progressive disease, CHOP.

*Preparation of lymph node biopsies*

Biopsies arrived in a fresh state. Part of the material was fixed in neutral buffered formalin for routine histopathology (hematoxylin–eosin, Giemsa, PAS and Laidlaw stains), and for silver staining in order to assess the mitotic index (Rehn *et al.*, 1991); when necessary, immunohistochemical staining of cytoplasmic immunoglobulins (Martinsson *et al.*, 1985) was undertaken. Another portion of the material was used for the preparation of a suspension of cells by mincing the tissue through a stainless-steel mesh. Part of this cell suspension was used for immunological phenotyping; part of it was frozen in liquid nitrogen and later used for measurements of the S-phase fraction (Rehn *et al.*, 1990*a*) and C-TK.

Cells to be used for the C-TK measurements were taken from the liquid nitrogen and thawed in a 37°C water bath, rinsed at 37°C in culture medium (RPMI + 10% fetal calf serum + 1% penicillin + glutamine) and then resuspended in phosphate-buffered saline. The cell number and viability of the cells (trypan blue exclusion method) was determined. After centrifugation, the cells were resuspended in a buffer containing Hepes 25 mM (pH 7.4), magnesium sulphate 2 mM and bovine albumin 2 mg ml$^{-1}$. Each preparation was divided into at least two samples and refrozen at −70°C.

*Assay of TK activity*

Determinations of TK levels in serum and cell suspensions were basically performed according to Gronowitz *et al.* (1984). The assay is based on the use of [$^{125}$I]iododeoxyuridine (IUdR) as a substrate and is available as a kit (Sangtec Medical, Bromma, Sweden). All TK values are given as units μl$^{-1}$, where 1 unit corresponds to a substrate turnover of $1.2 \times 10^{-18}$ katal. The TK activity was determined directly in undiluted serum, according to the kit insert. The upper normal limit of S-TK in healthy subjects is 5 units μl$^{-1}$.

Determination of TK activity in frozen cell suspensions was performed, as follows, in order to control the linearity of the enzyme reaction with time and sample dilution. Frozen suspensions were thawed, vortexed for 30 s and serially diluted in an ice-bath in five steps in a buffer containing Hepes 25 mM (pH 7.4), magnesium sulphate 2 mM, β-lactoglobulin 2 mg ml$^{-1}$ and glycerol (25% (v/v). From each dilution 80 μl was transferred to a new tube, whereafter the assay was started by adding 2 ml of reaction solution and transferring the tubes to 37°C. The amount of product formed was determined after 1, 2 and 3 h of incubation, by transferring 500 μl samples to new tubes containing the separator. These tubes were further processed according to the kit insert. The TK activity in each sample was calculated from the dilutions giving the linear turnover in relation to time and sample amount. All TK values given refer to at least two independent determinations on two different occasions, giving similar results (± 15%). The TK activity in one million cells was calculated.

*S-phase fraction and mitotic index*

Estimations of the S-phase fraction and mitotic index were performed as previously described (Rehn *et al.*, 1990*a*, 1991).

*Tumour volume calculation*

A more extended estimation of the tumour burden than that provided by the clinical stage was performed retrospectively by calculating the tumour volume (in cm$^3$) from data in the patient files of findings from clinical examinations, radiological examinations (chest radiography, ultrasonography, computerised tomography or magnetic resonance imaging of the abdomen and, in some cases, of the thorax) and bone marrow examinations (aspirations from the sternum and core biopsies from the pelvic bones) which revealed the cellularity and the degree of lymphoma involvement. Clinical examination, chest radiography, at least one radiological abdominal examination and bone marrow examination were done in all patients. If the clinical records did not provide a distinct assessment of the size of any tumour manifestation, the X-rays or bone marrow aspirations were re-examined. The entire bone marrow volume was estimated to 2600 cm$^3$, half of which was estimated to be red bone marrow (Block, 1976). Only the proportion of marrow replaced with tumour cells was included in the tumour volume.

The estimated tumour volumes were grouped into three categories: small (< 50 cm$^3$), medium (50–500 cm$^3$) and large (> 500 cm$^3$).

*Statistical methods*

Differences in the distribution of values between two groups were tested with the Mann–Whitney *U*-test, and differences in the distribution of values for several subgroups were tested with the Kruskal–Wallis test. The correlation between different parameters was done with Spearman rank correlation test. These tests and the multiple regression calculations were performed with StatView 4.0 (Abacus Concepts, 1992).

LIFETEST was used to evaluate the prognostic capacity of the different variables (SAS Institute, 1985). The log-rank test (Peto *et al.*, 1976) was used. Patients dying of intercurrent diseases were not included in the population at risk after their death, provided they were in complete clinical remission. Best cut-off points were defined as the level yielding the highest $\chi^2$-value, when equality over strata was tested with the log-rank test, provided that at least 15% of the cases had a value neither below nor above the cut-off level. The parameters were also tested as continuous variables. Multivariate analyses with the Cox's proportional hazards model were performed with Statistica 3.0b software (Statsoft, 1993). Chi-square and *P*-values in the multivariate analyses were obtained by Wald's test.

### Results

*C-TK in relation to S-phase fraction, mitotic index, histopathology and tumour volume*

A correlation was seen between C-TK and the two proliferation-associated parameters, S-phase fraction ($r = 0.6$, $P = 0.0001$) and mitotic index ($r = 0.6$, $P = 0.0001$). The correlatin between S-phase fraction and mitotic index was, however, somewhat higher ($r = 0.8$, $P = 0.0001$).

High-grade NHL tumours had significantly higher C-TK values (Table I and II), S-phase fractions and mitotic indices than low-grade NHL ($P = 0.0001$ in all cases).

Small and medium-sized tumours also had significantly higher values of C-TK ($P = 0.002$) (Table II), S-phase fraction ($P = 0.003$) and mitotic index ($P = 0.01$) than large tumours. An inverse correlation between C-TK and the numerical value of the tumour volume was seen in all patients and in high-grade NHL ($r = -0.4$, $P = 0.0001$, *vs* $r = -0.3$, $P = 0.03$). No correlation, however, was observed in the case of low-grade NHL ($r = -0.1$, $P = 0.65$).

When the C-TK levels of the four groups formed from low- or high-grade NHL together with 'small and medium-sized tumours' or 'large tumours' were compared, differences between the groups were seen ($P = 0.0003$, Figure 1a). In contrast, when the C-TK values were calculated per cell in

**Table I** Patient characteristics and mean values of tumour volume (cm³), S-TK (units μl⁻¹) and C-TK (units 10⁻⁶ cells) within the different subgroups of NHL according to the Kiel classification

| Histology | n | B symptoms n (%) | Stage I n | Stage II n | Stage III n | Stage IV n | Age (Years) Mean Range | S-TK Mean | Tumour volume Mean | C-TK Mean |
|---|---|---|---|---|---|---|---|---|---|---|
| Low grade | | | | | | | | | | |
| CLL | 20 | 3 (15) | 0 | 0 | 1 | 19 | 65 (43-84) | 15.4 | 1628 | 965 |
| IC | 7 | 2 (29) | 0 | 1 | 0 | 6 | 64 (35-85) | 8.0 | 1273 | 914 |
| CC | 2 | 1 (50) | 0 | 0 | 0 | 2 | 66 (53-78) | 13.3 | 939 | 4425 |
| fCB-CC | 14 | 5 (36) | 2 | 1 | 3 | 8 | 59 (40-81) | 9.2 | 1099 | 1571 |
| fd CB-CC | 5 | 1 (20) | 0 | 3 | 0 | 2 | 55 (42-71) | 11.2 | 896 | 3510 |
| All | 48 | 12 (25) | 2 | 5 | 4 | 37 | 62 (35-85) | 12.0 | 1317 | 1544 |
| High grade | | | | | | | | | | |
| CB | 26 | 10 (39) | 4 | 3 | 8 | 11 | 61 (25-77) | 21.4 | 424 | 11213 |
| IB | 6 | 3 (50) | 2 | 0 | 3 | 1 | 68 (37-87) | 20.2 | 500 | 4796 |
| LB | 5 | 4 (80) | 1 | 0 | 1 | 3 | 39 (12-66) | 47.1 | 259 | 16160 |
| Unclassif. | 4 | 3 (75) | 0 | 1 | 1 | 2 | 59 (39-68) | 73.1 | 788 | 4850 |
| All | 41 | 20 (49) | 7 | 4 | 13 | 17 | 59 (12-87) | 29.4 | 450 | 10256 |
| All | 89 | 32 (36) | 9 | 9 | 17 | 54 | 61 (12-87) | 20.0 | 918 | 5557 |

CLL, chronic lymphocytic leukaemia; IC, immunocytic; CC, centrocytic; fCB-CC, follicular centroblastic-centrocytic; fd CB-CC, follicular and diffuse centroblastic-centrocytic; CB, centroblastic-centrocytic; IB, immunoblastic; LB, lymphoblastic; Unclassif., unclassifiable lymphoma.

**Table II** TK levels in serum (units μl⁻¹) and tumour cells (units 10⁻⁶ cells) in relation to histological group and tumour volume category

| Histology | Tumour volume | n | S-TK Mean | S-TK Median | S-TK Range | C-TK Mean | C-TK Median | C-TK Range | Correlation S-TK/C-TK r | P |
|---|---|---|---|---|---|---|---|---|---|---|
| Low grade | Small | 4 | 3.0 | 2.9 | 1.6-4.8 | 2825 | 575 | 100-10050 | 0.8 | NS |
| | Medium | 7 | 5.2 | 3.4 | 1.0-11.3 | 2136 | 1500 | 550-4600 | 0.5 | NS |
| | Large | 37 | 14.2 | 8.6 | 2.6-77.0 | 1293 | 650 | 50-8300 | 0.3 | NS |
| | All | 48 | 12.0 | 6.6 | 1.0-77.0 | 1544 | 675 | 50-10050 | 0.2 | NS |
| High grade | Small | 9 | 3.7 | 3.9 | 1.8-5.0 | 12583 | 14775 | 75-28725 | 0.5 | NS |
| | Medium | 21 | 14.4 | 9.8 | 4.2-35.0 | 12915 | 7350 | 750-44700 | 0.2 | NS |
| | Large | 11 | 79.1 | 51.4 | 6.2-272.0 | 3275 | 3450 | 250-7650 | 0.2 | NS |
| | All | 41 | 29.4 | 9.8 | 1.8-272.0 | 10256 | 5250 | 75-44700 | −0.1 | NS |
| All | Small | 13 | 3.5 | 3.8 | 1.6-5.0 | 9581 | 4350 | 75-28725 | 0.6 | <0.05 |
| | Medium | 28 | 12.1 | 8.8 | 1.0-35.0 | 10220 | 5113 | 550-44700 | 0.4 | <0.05 |
| | Large | 48 | 29.1 | 11.1 | 2.6-272.0 | 1747 | 800 | 50-8300 | 0.4 | <0.01 |
| | All | 89 | 20.0 | 20.1 | 1.0-272.0 | 5557 | 1800 | 50-44700 | 0.2 | NS |

*r*, Spearman correlation coefficient; NS, not significant.

mitosis or per cell in S-phase, more homogeneous levels were observed between the four groups (Figure 1b and c) with no statistically significant differences between the groups ($P = 0.3$ *vs* $P = 0.2$). High-grade NHL did not have higher C-TK levels per cell during mitosis or S-phase than low-grade NHL. Similarly, the C-TK values per cell in mitosis did not differ between 'small and medium-sized tumours' and 'large tumours', whereas a borderline significant difference ($P = 0.05$) was seen in the C-TK values per cell in S-phase between 'small and medium-sized tumours' and 'large tumours'.

*Tumour volume in relation to stage and histopathology*

There was a significant difference in tumour volume between the different Ann Arbor stages ($P = 0.0001$), although values overlapped, especially among stages II and III (data not illustrated).

Low-grade NHL had significantly higher tumour volumes than high-grade NHL ($P = 0.0001$) (Table I).

*S-TK in relation to histopathology, tumour volume and stage*

High-grade NHL had higher S-TK values than low-grade NHL ($P = 0.03$) (Tables I and II).

A correlation between S-TK and the numerical value of tumour volume was seen in all patients ($r = 0.4$, $P = 0.0001$) and in both low-grade NHL ($r = 0.6$, $P = 0.0001$) and high-grade NHL ($r = 0.8$, $P = 0.0001$).

The levels of S-TK differed significantly between the different Ann Arbor stages ($P = 0.004$; Figure 2a) and the estimated tumour volume categories ($P = 0.0001$; Figure 2b).

*S-TK in relation to tumour volume and C-TK*

Among all patients, there was no correlation between S-TK and C-TK ($r = 0.2$, $P = 0.06$) (Figure 3). All patients with high S-TK values (>35 units μl⁻¹) had low or moderately elevated C-TK values (mean 2902 units 10⁻⁶ cells, range 150-7650 units 10⁻⁶ cells) whereas all patients with high C-TK values (>10000 units 10⁻⁶ cells) had low or moderately elevated S-TK values (mean 12.1 units μl⁻¹, range 3.1-35.0 units μl⁻¹). The former patients had high tumour volumes (mean 1673 cm³, range 667-3185 cm³), whereas the latter had low tumour volumes (mean 107 cm³, range 8-325 cm³). Patients with both comparatively low S-TK and C-TK values had an intermediate mean tumour volume (993 cm³) but this varied greatly (2-3094 cm³).

In contrast to the lack of correlation between S-TK and C-TK in all patients, a correlation between S-TK and C-TK

was seen within the three tumour volume categories (Table II). This correlation was not seen within the different stages according to Ann Arbor (data not illustrated). Within the different tumour volume categories, the levels of S-TK varied according to whether C-TK levels were above or below the median value (Table III). Multiple regression analyses were performed to evaluate the relative importance of tumour volume and C-TK for the S-TK level. The values were then used in a logarithmic form in order to correct for different scaling. Both tumour volume and C-TK gave significant contribution to the variations in the S-TK level and the following relationship was found:

$$\text{Log S-TK} = -0.087 + 0.234 \text{ (log tumour volume)} + 0.171 \text{ (log C-TK)}$$

The equation shows that tumour volume had the strongest relationship to the S-TK level ($P = 0.0001$). After the effect of the tumour volume was taken into account, C-TK also contributed to the S-TK level ($P = 0.009$).

### *B symptoms in relation to histopathology, tumour volume and TK levels*

Sixty-three per cent (20/32) of the patients with B symptoms had high-grade NHL. More high-grade NHL patients than low-grade NHL patients ($P = 0.02$) (Table I) had B symptoms.

**Figure 1** (**a**) C-TK (units $10^{-6}$ cells) in patients with low- and high-grade NHL with small or medium-sized (SM) or large (L) tumours. Only the 77 patients in whom S-phase and mitotic index values were also available are included. (**b**) C-TK/mitosis [units $10^{-6}$ cells divided by the mean number of mitosis in ten high-power fields ($\times$ 40), area 0.055 mm$^2$] and (**c**) C-TK/S-phase (units $10^{-6}$ cells divided by the percentage of cells in S-phase) for the same groups. Mean values are indicated together with the 95% confidence intervals.

No patient within the small tumour volume category had B symptoms, whereas almost half of the patients with medium and large tumour volumes had B symptoms (data not illustrated).

Patients with B symptoms had significantly higher S-TK levels ($P = 0.002$) and slightly higher C-TK values ($P = 0.03$) than patients without B symptoms. Of the patients with medium and large tumour volumes, the patients with B symptoms had much higher C-TK values (mean 6982 units $10^{-6}$ cells) than patients without B symptoms (mean 3332 units $10^{-6}$ cells, $P = 0.007$).

### *Relations to prognosis*

S-TK, C-TK and tumour volume all carried prognostic information for the patient sample as a whole (Figure 4), in patients with low-grade NHL and, with the exception of C-TK, in patients with high-grade NHL (data not illustrated). The separation of the variables into two prognostic groups is in the case of C-TK, most useful after a short-term follow-up, and in the case of tumour volume after a long-

**Figure 2** S-TK (units $\mu l^{-1}$) in different clinical stages (**a**) and tumour volume categories (**b**). Mean values are indicated together with the 95% confidence intervals.

**Figure 3** Relation between S-TK (units $\mu l^{-1}$) and C-TK (units $10^{-6}$ cells). The dotted lines indicate the limits of 'high' S-TK and C-TK values.

Table III Mean and median S-TK values in the different tumour volume groups according to whether C-TK levels were below or above the median C-TK value

| Tumour volume | C-TK<1800 S-TK Mean | Median | n | C-TK≥1800 S-TK Mean | Median | n | P-value[a] |
|---|---|---|---|---|---|---|---|
| Small ($n = 13$) | 2.5 | 2.0 | 5 | 4.2 | 4.6 | 8 | 0.02 |
| Medium ($n = 28$) | 7.0 | 5.1 | 7 | 13.8 | 9.0 | 21 | 0.08 |
| Large ($n = 48$) | 16.0 | 8.6 | 32 | 55.3 | 27.4 | 16 | 0.003 |
| ($P$-value[b]) | | 0.006 | | | 0.0001 | | |

[a]Difference in S-TK according to C-TK level. [b]Difference in S-TK between tumour volume categories.

Figure 4 Probability of survival in all patients with NHL according to (a) S-TK levels [S-TK>20 units $\mu l^{-1}$ ($n = 22$); S-TK<20 units $\mu l^{-1}$ ($n = 67$), log-rank $P = 0.0001$], (b) C-TK levels [C-TK>2550 units $10^{-6}$ cells ($n = 38$); C-TK<2550 units $10^{-6}$ cells ($n = 51$), log-rank $P = 0.02$] and (c) tumour volumes [>1200 cm$^3$ ($n = 30$); <1200 cm$^3$ ($n = 59$), log-rank $P = 0.01$].

term follow-up, whereas S-TK separates the prognostic groups after both a short- and long-term follow-up (Figure 4).

Stage also had prognostic value (log-rank $P = 0.03$), whereas histological grade provided no prognostic information (log-rank $P = 0.20$). In multivariate analyses involving S-TK, C-TK and tumour volume (or stage) and the histological grade, S-TK showed superior prognostic strength and no additional information was provided by any of the other parameters (data not illustrated).

Table IV Independent relations to prognosis of S-TK, C-TK and tumour volume; results of multivariate analyses using the variables in continuous form

| Variable | $\beta$ | s.e. ($\beta$) | $\chi^2$ | P |
|---|---|---|---|---|
| *(a) All three variables included* | | | | |
| S-TK | 0.0091576 | 0.0031920 | 8.23 | <0.01 |
| C-TK | 0.0000343 | 0.0000189 | 3.29 | NS |
| Volume | 0.0002621 | 0.0001843 | 2.02 | NS |
| *(b) Only two variables included* | | | | |
| S-TK | 0.0107534 | 0.0030046 | 12.81 | <0.001 |
| C-TK | 0.0000230 | 0.0000173 | 1.77 | NS |
| S-TK | 0.0098184 | 0.0032067 | 9.37 | <0.01 |
| Volume | 0.0001373 | 0.0001718 | 0.64 | NS |
| C-TK | 0.0000372 | 0.0000184 | 4.09 | <0.05 |
| Volume | 0.0003617 | 0.0001715 | 4.45 | <0.05 |

NS, not significant.

In order to explore further the relations between S-TK, C-TK and tumour volume, their association to prognosis was tested in separate multivariate analyses including all three variables or only two of them. It was found that neither tumour volume nor C-TK gave any prognostic information additional to S-TK (Table IV). In the absence of S-TK, C-TK and tumour volume each provided additional prognostic information (Table IV). Using log-transformed data or using the vairables in dichotomised form did not change the results (data not illustrated).

### Discussion

The prognostic value of S-TK in patients with NHL is well established (Ellims *et al.*, 1981; Gronowitz *et al.*, 1983; Hagberg *et al.*, 1984*a*; Martinsson *et al.*, 1988; Rehn *et al.*, 1991). It has been suggested that S-TK reflects both tumour cell proliferation rate and tumour volume (Rehn *et al.*, 1991; van der Gaast *et al.*, 1991; Luoni *et al.*, 1991), both of which are of prognostic importance. The results of this study strongly suggest that S-TK reflects the tumour volume in particular, but also the proliferation rate. In certain other tumour types, S-TK has also been found to be correlated to tumour burden and is elevated in higher stages [Simonsson *et al.*, 1988; Luoni *et al.*, 1991 (multiple myeloma); Eriksson *et al.*, 1985 (Hodgkin's disease); McKenna *et al.*, 1988; Robertsson *et al.*, 1991 (breast cancer); Gronowitz *et al.*, 1986; Lehtinen *et al.*, 1988; van der Gaast *et al.*, 1991 (small-cell lung cancer)].

The tumour volume was a good predictor of prognosis, whereas clinical stage was of less prognostic importance. It is known that the prognostic importance of the Ann Arbor stage, which was originally developed for HD, is not particularly strong in NHL (Leonard et al., 1983). Yet, staging according to Ann Arbor is in routine use as regards therapy decisions and is used for comparing results from different trials. This is probably because of its simplicity. Attempts to replace this staging system by other tumour burden assessments have failed to come in routine clinical use. Since the tumour bùrden assessment in this study was performed retrospectively, uncertainties may exist, and it is not our intention to advocate its routine use. However, we believe that it carries some validity, particularly after categorisation into small, medium and large volumes, in the exploration of the importance of tumour burden as regards S-TK levels.

C-TK correlated well with other proliferation-associated factors, supporting the idea based upon theoretical considerations that the level of C-TK reflects proliferation. S-phase fraction and mitotic index are also, like C-TK, significantly higher in high-grade NHL than in low-grade NHL. Interestingly, although patient numbers were small within the low-grade NHL group, the highest C-TK levels were found in the two groups with an intermediate prognosis, namely centrocytic lymphomas and follicular and diffuse centroblastic–centrocytic lymphomas (Martinsson et al., 1988). The C-TK level per cell in mitosis (or per cell in S-phase) in patients with high- and low-grade NHL with 'small and medium sized' or 'large' tumour volumes, respectively, did not differ significantly between the groups, indicating that the content of C-TK in cells in which C-TK is expressed (S-phase, $G_2$ or mitosis) is very much the same despite the large variability in proliferation rates.

Even if there was no correlation between S-TK and C-TK in the patient sample as a whole, the correlations between these parameters seen within each tumour volumes group suggest that the S-TK level depends not only upon the tumour volume, but also upon the cell content of TK, and thus also reflects cell proliferation. Also, despite significantly larger tumour volumes in the low-grade NHL group *vis-à-vis* the high-grade NHL, S-TK was significantly higher in patients with high-grade NHL than low-grade NHL. Multiple regression analyses showed that tumour volume had the strongest relationship to the S-TK level but that C-TK provided additional information after the tumour volume was taken into account. Further support for the importance of both C-TK and tumour volume as regards the levels of S-TK comes from multivariate analyses, in which C-TK and tumour volume showed additional prognostic importance but neither C-TK, nor tumour volume added any significant information to that provided by S-TK.

The finding that the C-TK values were higher in patients with small or medium-sized tumours ($<500\ cm^3$) than in patients with large tumours probably reflects the fact that rapidly proliferating tumours become symptomatic much earlier than slowly proliferating ones. In fact, not a single patient with NHL had both high C-TK and high S-TK levels at diagnosis. In patients with acute lymphatic leukaemia, which is usually a highly proliferative disease, very high S-TK level may be seen (Hagberg et al., 1984c). The C-TK levels in the tumour cells of patients with ALL collected *in vivo* showed levels of the enzyme as high as in high-grade NHL (Vertongen et al., 1984). It is known that the tumour volumes in patients with acute leukaemia are generally higher than in patients with the closely related lymphoblastic lymphoma. Untreated patients suffering from acute leukaemias have a very short survival, indicating that both high cell proliferation rate (high C-TK) and a large tumour burden are incompatible with prolonged life.

This study does not explore how cellular TK reaches the blood, although one possible explanation may be through cell death. We have, in a number of patients with NHL, seen a significant increase in S-TK during the days immediately after chemotherapy administration, with peak levels after 24–48 h (own unpublished observations). The half-life of S-TK has been estimated to be less than 2 days (Gronowitz and Källander, 1984). Catalano et al. (1990) have also shown an increase in S-TK 12–48 h after intensive chemotherapy in patients with acute myelogenous leukaemia with a reduction or normalisation, parallel with the blast cell disappearance in blood, during the following days. Elevated S-TK values are seen in patients with megaloblastic anaemia as a result of vitamin $B_{12}$ deficiency (Hagberg et al., 1984c). In that condition, enhanced TK values are also found in the bone marrow (Nakao et al., 1968), and it is proposed that the haemolysis of proliferating immature cells gives rise to the S-TK elevation (Hagberg et al., 1984c). We therefore suggest that the level of TK in serum reflects to a great extent the number of proliferating cells that have died within a few days of the sampling, even if a release of TK from 'healthy' proliferating cells cannot be excluded. Bristow et al. (1988) in fact showed that proliferating cells in culture release TK into the surrounding medium. In studies of liver regeneration in rats, S-TK and C-TK rise simultaneously (Polimeno et al., 1991). An elevation of S-TK after liver resections is also seen in humans (Francavilla et al., 1990).

In tumour sections, areas of tumour cell necrosis are seen in high-grade NHL but rarely in low-grade NHL (own unpublished observations). A heterogeneous appearance, when investigated with magnetic resonance imaging (MRI), of high-grade NHL, as opposed to low-grade NHL, is most likely a reflection of tumour cell necrosis (Rehn et al., 1991), observations indicating cell death in rapidly proliferating tumours. Preliminary analyses have shown that patients with stage I disease (often low tumour volume) have a good prognosis despite the MRI appearance (homogeneous or heterogeneous), whereas, in stages II–IV, the prognosis is poorer for heterogeneous tumours (Rehn et al., 1991). These results thus also fit in with our suggestion.

In conclusion, this study provides evidence that the levels of TK in serum depend both on the tumour burden and upon the cellular content of TK, i.e. cell proliferation. This fact may explain TK's strong prognostic importance in patients with malignant lymphomas and why it is superior to most other strong predictors in a number of studies (Hagberg et al., 1984a; Martinsson et al., 1988; Rehn et al., 1991).

**Acknowledgements**

This work was supported by grants from the Swedish Cancer Society, (Project Numbers 1937-B93-10XCC and 1942-B93-11XBC). The technical assistance of Anneli Kraft and Ulla Långström-Persson is gratefully acknowledged.

### References

ABACUS CONCEPTS. (1992). *StatView™ 4.0*. Abacus Concepts: Berkeley, CA.

ÅKERMAN M, BRANDT L, JOHNSSON A AND OLSSON H. (1987). Mitotic activity in non-Hodgkin's lymphomas. Relation to the Kiel classification and to prognosis. *Br. J. Cancer*, **55**, 219–223.

ARCHIMBAUD E, VIGREUX B, TIGAUD J-D, MAUPAS J, GUYOTAT D, VIALA J-J AND FIERE D. (1988). Serum thymidine kinase in acute nonlymphoblastic leukemia. *Leukemia*, **2**, 245–246.

BRISTOW H, O'NEILL K, HANNIGAN BM AND MCKENNA PG. (1988). Leakage of thymidine kinase from proliferating cells. *Biochem. Soc. Trans.*, **16**, 55–56.

BLOCK M. (1976). *Text–Atlas of Hematology*. Lea and Febiger: Philadelphia.

CARBONE PP, KAPLAN HS, MUSSHOFF K, SMITHERS DW AND TUBIANA M. (1971). Report of the Committee in Hodgkin's disease staging classification. *Cancer Res.*, **31**, 1860–1861.

CATALANO L, FRIGERI F, CAMERA A, DE ROSA G, FESTINESE R AND ROTOLI B. (1990). Kinetics of serum TK and LDH during therapy for AML. *Haematologica*, **75**, 301–303.

DONHUIJSEN K. (1987). Mitosis in non-Hodgkin's lymphomas, frequency and prognostic relevance. *Pathol. Res. Pract.*, **182**, 352–357.

ELLIMS P, ENG GAN T, MEDLEY G AND VAN DER WEYDEN MB. (1981). Prognostic relevance of thymidine kinase isozymes in adult non-Hodgkin's lymphoma. *Blood*, **58**, 926–930.

ENG GAN T, FINCH PD, BRUMLEY JL, HALLAM LJ AND VAN DER WEYDEN MB. (1984). Pyrimidine and purine activities in non-Hodgkin's lymphoma. Correlation with histological status and survival. *Eur. J. Cancer Clin. Oncol.*, **20**, 361–368.

ERIKSSON B, HAGBERG H, GLIMELIUS B, SUNDSTRÖM C, GRONOWITZ S AND KÄLLANDER C. (1985). Serum thymidine kinase as a prognostic marker in Hodgkin's disease. *Acta Radiol. Oncol.*, **24**, 167–171.

FRANCAVILLA A, PANELLA C, POLIMENO L, GIANGASPERO A, MAZZAFERRO V, PAN C-E, VAN THIEL D AND STARZL TE. (1990). Hormonal and enzymatic parameters of hepatic regeneration in patients undergoing major liver resection. *Hepatology*, **12**, 1134–1138.

VAN DER GAAST A, VAN PUTTEN WLJ, OOSTEROM R, COZIJNSEN M, HOEKSTRA R AND SPLINTER TAW. (1991). Prognostic value of serum thymidine kinase, tissue polypeptide antigen and neuron specific enolase in patients with small cell lung cancer. *Br. J. Cancer*, **64**, 369–372.

GRONOWITZ S AND KÄLLANDER C. (1984). Extracellular expression of TK isoenzymes in human body fluids, with special reference to herpes virus diagnostics and use for monitoring of antiviral therapy. In *New Horizons in Microbiology*, Sanna A and Morace G (eds) pp. 273–284. Elsevier Science Publishers: Amsterdam.

GRONOWITZ S, HAGBERG H, KÄLLANDER C AND SIMONSSON B. (1983). The use of serum deoxythymidine kinase as a prognostic marker, and in the monitoring of patient with non-Hodgkin's lymphoma. *Br. J. Cancer*, **47**, 487–495.

GRONOWITZ S, KÄLLANDER C, HAGBERG H, DIDERHOLM H AND PETTERSSON U. (1984). Application of an in vitro assay for serum thymidine kinase: results on viral disease and malignancies in humans. *Int. J. Cancer*, **33**, 5–12.

GRONOWITZ S, STEINHOLZ L, KÄLLANDER C, HAGBERG H AND BERGH J. (1986). Serum thymidine kinase in small cell cancer of the lung: relation to clinical features, prognosis, and other biochemical markers. *Cancer*, **58**, 111–118.

HAGBERG H, GLIMELIUS B, GRONOWITZ S, KILLANDER A, KÄLLANDER C AND SCHRÖDER T. (1984*a*). Biochemical markers in non-Hodgkin's lymphoma stage III and IV and prognosis: a multivariate analysis. *Scand. J. Haematol.*, **33**, 59–67.

HAGBERG H, GRONOWITZ S, KILLANDER A AND KÄLLANDER C. (1984*b*). Serum thymidine kinase in vitamin $B_{12}$ deficiency. *Scand. J. Haematol.*, **32**, 41–45.

HAGBERG H, GRONOWITZ S, KILLANDER A, KÄLLANDER C, SIMONSSON B, SUNDSTRÖM C AND ÖBERG G. (1984*c*). Serum thymidine kinase in acute leukemia. *Br. J. Cancer*, **49**, 537–540.

HAGBERG H, LINDEMALM C AND CAVALLIN-STÅHL E FOR THE SWEDISH LYMPHOMA STUDY GROUP. (1988). CHOP versus CHOP-M in the treatment of high grade malignant non-Hodgkin's lymphomas in adults: a Swedish national randomized study. *Proc. ASCO*, **7**, 243.

KIMBY E, BJÖRKHOLM M, GAHRTON G, GLIMELIUS B, HAGBERG H, JOHANSSON B, JOHANSSON H, JULIUSSON G, JÄRNMARK M, LÖFVENBERG E, KILLANDER A, LERNER R, LINDEMALM C, PETTERSSON U, ROBERT K-H, SIMONSSON B, STALFELT A-M, SUNDSTRÖM C, SVEDMYR B, UDÉN A-M, WADMAN B, WAHLIN A, ÖST Å AND MELLSTEDT H. (1994). Chlorambucil/prednisolone vs. CHOP in symptomatic low-grade non-Hodgkin's lymphomas: a randomised trial from the Lymphoma Group of Central Sweden. *Ann. Oncol.*, **5** (Suppl. 2) 67–71.

KLIMO P AND CONNORS J. (1985). MACOP-B chemotherapy for the treatment of diffuse large-cell lymphoma. *Ann. Intern. Med.*, **102**, 596–602.

LEHTINEN M, WIGREN T, LEHTINEN T, KALLIONIEMI O-P, AINE R, ARRAN R-K AND OJALA A. (1988). Correlation between serum tumor marker levels and tumor proliferation in small cell lung cancer. *Tumour Biol.*, **9**, 287–292.

LEONARD R, CUZICK J, MACLENNAN I, VANHEGAN R, MACKIE P, MCCORMICK C AND OXFORD LYMPHOMA GROUP. (1983). Prognostic factor in non-Hodgkin's lymphoma: the importance of symptomatic stage as an adjunct to the Kiel histopathological classification. *Br. J. Cancer*, **47**, 91–102.

LENNERT K. (1978). *Malignant Lymphomas other than Hodgkin's Disease*. Springer: Berlin.

LEWENHAUPT A, EKMAN P, ENEROTH P AND NILSSON B. (1990). Tumour markers as prognostic aids in prostatic carcinoma. *Br. J. Urol.*, **66**, 182–187.

LUONI R, UCCI G, RICCARDI A, GOBBI P, AVATO FM, VIGNALE C AND ASCARI E FOR THE COOPERATIVE GROUP FOR STUDY AND TREATMENT OF MULTIPLE MYELOMA. (1992). Serum thymidine kinase in monoclonal gammopathies, a prospective study. *Cancer*, **69**, 1368–1372.

MARTINSSON U, GLIMELIUS B, HAGBERG H, SIMONSSON B AND SUNDSTRÖM C. (1985). Intracytoplasmic immunoglobulins in the differential diagnosis of lymphocytic lymphomas of the B-cell type and immunocytic lymphomas. *Acta Radiol. Oncol.*, **24**, 527–535.

MARTINSSON U, GLIMELIUS B, HAGBERG H AND SUNDSTRÖM C. (1988). Prognostic relevance of serum-markers in relation to histopathology, stage and initial symptoms in advanced low-grade non-Hodgkin lymphomas. *Eur. J. Haematol.*, **40**, 289–298.

MCKENNA PG, O'NEILL KL, ABRAM WP AND HANNIGAN BM. (1988). Thymidine kinase activities in mononuclear leucocytes and serum from breast cancer patients. *Br. J. Cancer*, **57**, 619–622.

NAKAO K AND FUJIOKA S. (1968). Thymidine kinase activity in the human bone marrow from various blood diseases. *Life Sci.*, **7**, 395–399.

PETO R, PIKE M, ARMITAGE P, BRESLOW N, COX D, HOWARD S, MANTLE N, MCPHERSON K, PETO J AND SMITH P. (1976). Design and analysis of randomised clinical trials requiring prolonged observation of each patient. I. Introduction and design. *Br. J. Cancer*, **34**, 585–612.

POLIMENTO L, AZZARONE A, DELL'AQUILA P, AMORUSO C, BARONE M, ANGELINI A, VAN THIEL DH AND FRANCAVILLA A. (1991). Relationship between plasma and hepatic cytosolic levels of ornithine decarboxylase (ODC) and thymidine kinase (TK) in 70% hepatectomized rats. *Dig. Dis. Sci.*, **36**, 289–292.

REHN S, GLIMELIUS B, STRANG P, SUNDSTRÖM C AND TRIBUKAIT B. (1990*a*). Prognostic significance of flow cytometry studies in B-cell non-Hodgkin lymphomas. *Hematol. Oncol.*, **8**, 1–12.

REHN S, NYMAN R, GLIMELIUS B, HAGBERG H AND SUNDSTRÖM C. (1990*b*). Magnetic resonance imaging for predicting prognostic grade in non-Hodgkin lymphomas. *Radiology*, **176**, 249–253.

REHN S, GLIMELIUS B AND SUNDSTRÖM C. (1991). A comparative study of proliferation-associated parameters in B-cell non-Hodgkin lymphomas. *Hematol. Oncol.*, **9**, 287–298.

ROBERTSSON JFR, O'NEILL KL, THOMAS MW, MCKENNA PG AND BLAMEY RW. (1990). Thymidine kinase in breast cancer. *Br. J. Cancer*, **62**, 663–667.

ROMAIN S, JAVRE JL, SAMPEREZ S, JOUAN P, BRESSAC C, VARETTE I, BRANDONE H AND MARTIN PM. (1990). Valeur pronostique de la thymidine kinase dans le cancer du sein. *Bull. Cancer*, **77**, 973–983.

SAS INSTITUTE. (1985). *SAS User's Guide: Statistics*, Version 5 Edition. SAS Institute: Cary, NC.

SIMONSSON B, KÄLLANDER C, BRENNING G, KILLANDER A, GRONOWITZ J, BERGSTRÖM R AND ÅHRE A. (1988). Biochemical markers in multiple myeloma: a multivariate analysis. *Br. J. Haematol.*, **69**, 47–53.

STATSOFT. (1993). *Statistica™ 3.0b*. StatSoft: Tulsa, OK.

SHERLEY J AND KELLEY T. (1988). Regulation of human thymidine kinase during the cell cycle. *J. Biol. Chem.*, **263**, 8350–8358.

TUBIANA M, CARDE P, BURGERS JM, COSSET JM, VAN GLABBEKE M AND SOMMERS R. (1986). Prognostic factors in non-Hodgkin lymphoma. *Int. J. Radiat. Oncol.*, **12**, 503–514.

VERTONGEN F, FONDU P, VAN DEN HEULE B AND MANDELBAUM IM. (1984). Thymidine kinase and thymidine phosphorylase activities in various types of leukaemia and lymphoma. *Tumor Biol.*, **5/6**, 303–311.

WOOLDRIGE TN, GRIERSSON HL, WEISENBURGER DD, ARMITAGE JO, SANGER WG, COLLINS MM, PIERSON JL, PAUZA ME, FORDYCE R AND PURTILO DT. (1988). Association of DNA content and proliferative activity with clinical outcome in patients with diffuse mixed cell and large cell non-Hodgkin's lymphoma. *Cancer Res.*, **48**, 6608–6613.

YOUNG G, HEDLEY D, RUGG C AND ILAND H. (1987). The prognostic significance of proliferative activity in poor histology non-Hodgkin's lymphoma: a flow cytometry study using archival material. *Cancer Clin. Oncol.*, **23**, 1497–1504.